# Description of *Medwayella independencia* (Siphonaptera, Stivaliidae), a new species of flea from Mindanao Island, the Philippines and their phoretic mites, and miscellaneous flea records from the Malay Archipelago

**DOI:** 10.3897/zookeys.408.7479

**Published:** 2014-05-13

**Authors:** Michael W. Hastriter, Sarah E. Bush

**Affiliations:** 1 Monte L. Bean Life Science Museum, Brigham Young University, Provo, Utah, U.S.A.; 2 Department of Biology, University of Utah, Salt Lake City, Utah 84112, U.S.A.

**Keywords:** Bat fleas, key, phoresy, *Psylloglyphus*, *Urogale everetti*, Winterschmidtiidae

## Abstract

*Medwayella independencia*, a new species of flea, is described from the tupaiid host *Urogale everetti* (Thomas) from Mindanao Island, Philippines. Several other species of fleas are also recorded from the Philippines including a single male of *Lentistivalius philippinensis* Hastriter and Bush, 2013 (previously known only from two males), the bat fleas *Thaumapsylla breviceps orientalis* Smit and *Thaumapsylla longiforceps* Traub, a single unidentified female species of *Macrostylophora* Ewing collected from the murid *Bullimus bagobos* Mearns, and a pair of *Medwayella robinsoni* ssp. from *Sundasciurus hoogstraali* (Sanborn) from Busuanga Island, Philippines. Representatives of *Medwayella* Traub, 1972 and *Macrostylophora* have not previously been recorded from the Philippines. A key to the male sex of *Medwayella* is provided. Phoretic mites of the genus *Psylloglyphus* (family Winterschmidtiidae) were present under the abdominal sclerites of several male and female specimens of *M. independencia*. This is the second report of a phoretic mite on a species of *Medwayella* Traub. The co-evolutionary implications between phoretic mites and fleas are discussed.

## Introduction

The genus *Medwayella* Traub, 1972 contains 22 species (including subspecies) distributed as follows: Cambodia (1), Java (2), Peninsular Malaysia (7), Sabah (4), Sarawak (5), Sulawesi (1), Sumatra (3), and Thailand (2) (some species are reported in multiple areas). The first of these species was described by Rothschild (1905), followed by four additional species by Jordan (1926, 1933). Subsequently, Traub (1972a) erected the genus *Medwayella*, placed the previously described taxa by Rothschild and Jordan into *Medwayella*, and described an additional 13 new *Medwayella* species. Traub (1972a) also provided a detailed analysis of host flea relationships, a complex list of morphological characters germane to the genus, and a key to *Medwayella*. *Stivalius cambodius* Klein, 1970 was later included as a member of *Medwayella* by Mardon (1978). Mardon (1981) summarized the species housed in the British Museum while Beaucournu and Wells (2004) added another three species from Sabah and Durden and Beaucournu (2006) described one new taxon from Sulawesi. A small series of fleas from Mindanao, Philippines contained a new species of *Medwayella* that is described herein bringing the total number of species of *Medwayella* to 23. This new species also harbored phoretic mites and these are discussed.

## Materials and methods

Mammals and their ectoparasites were surveyed on Mindanao Island, Philippines, during June and July of 2012. Mammals were captured and euthanized according to guidelines of the American Society of Mammalogists (Gannon et al. 2007). Mist nets and harp traps were set in the forest and at, or near cave entrances to capture bats. Bats were processed for ectoparasites in accordance with Hastriter and Bush (2006). Terrestrial mammals were captured with Sherman traps or snap-traps. Each mammal was subjected to a thorough post-mortem visual examination: the face and ears were carefully searched and parasites were removed with forceps. In addition, the fur was systematically searched with the aid of a fine-toothed metal comb (LiceMeister®, National Pediculosis Association, Needham, MA). All ectoparasites recovered were preserved in 95% ethanol for later processing and identification in the laboratory. All associated hosts were prepared as museum specimens and were deposited in the Kansas Museum of Natural History (KUMNH), Lawrence, KS, U.S.A. Terminologies for anatomical terms of flea morphology follow those of Rothschild and Traub (1971) and Traub (1972a). Numbered tergites and sternites are designated with abbreviations as (T) and (S), e.g., T-II–V, and S-II–V. Repositories for fleas are designated as Carnegie Museum Natural History (CMNH), or Brigham Young University flea collection (BYUC). Images were prepared using an Olympus BX61 Compound Microscope, Olympus CC12 digital camera accompanied with an Olympus Microsuite™ B3SV program and Adobe Photoshop, CS4.

## Results

### Siphonaptera

#### Ceratophyllidae

##### Macrostylophora sp.

###### Material examined.

Philippines, Mindanao Island, village: San Antonio (09.0625°N, 125.6726°E), Mt. Hilong, 990 m, *Bullimus bagobos* Mearns ♀ (host # KUMNH-168368, NCA-179), 12 VI 2012, E. DiBlasi, 1♀ (parasite # P-5162) (BYUC).

###### Remarks.

This is the first representative of the genus *Macrostylophora* recorded from the Philippines. This single female likely represents an undescribed species; however, without additional material, especially males, it would be premature to describe the species at this time. Few ceratophyllid genera are found in Southeast Asia and the Philippines is the periphery of the genus *Macrostylophora*. Additional collecting is badly needed to better delineate the geographic parameters of *Macrostylophora* and this potentially new species of interest.

#### Ischnopsyllidae, Thaumapsyllinae

##### Thaumapsylla breviceps orientalis

Smit, 1954

###### Material examined.

Philippines, Luzon Island, Aurora Province, San Luis, Minoli, 15.680°N, 121.529°E, 520m, *Rousettus amplexicaudatus* (E. Geoffroy) ♂ (host# KUMNH-167960, JAE-3026F), J. Esselstyn, 16 VI 2009, 1♀ (parasite # JAE-3026) (BYUC).

###### Remarks.

The nominate subspecies is commonly found on *Rousettus aegyptiacus* (Geoffroy) in its western ranges from South Africa to Southwest Asia while in more eastern areas, *Thaumapsylla breviceps orientalis* is found on *Rousettus amplexicaudatus*. Other Philippine records of *Thaumapsylla breviceps orientalis* were discussed in Hastriter and Bush (2013).

##### Thaumapsylla longiforceps

Traub, 1951

http://species-id.net/wiki/Thaumapsylla_longiforceps\according to Hastriter et al 2014

###### Material examined.

Philippines, Mindanao Island, village: San Antonio (09.064°N, 125.642°E), Mt. Hilong, 110 m, *Rousettus amplexicaudatus* ♀ (host # KUMNH-168427, NCA-264), 20 VI 2012, E. DiBlasi, 1♀ (parasite #: P-5383) (BYUC).

###### Remarks.

This species commonly occurs on pteropodid bats (fruit bats) but has also been documented on vespertilionid and rhinolophid bats in Asia. See additional discussion of *Thaumapsylla longiforceps* in the Philippines by Hastriter and Bush (2013).

#### Stivaliidae, Stivaliinae

##### Lentistivalius philippinensis

Hastriter & Bush, 2013

http://species-id.net/wiki/Lentistivalius_philippinensis\according to Hastriter et al 2014

###### Material examined.

Sumatra, 6 km from Sidikalang, North Sumatra Province, Indonesia, “scrub and lalang grass near stream”; *Rattus rattus diardii* ♀, 8 IV 1973, M. Nadchatram, R. Traub, and D. Roberts, (B-87343, Sub. 231, 1♂) (CMNH).

###### Remarks.

Hastriter and Bush (2013) described this species from Luzon Island, Philippines, from *Crocidura grayi* Dobson. A single male was thereafter discovered in the Traub flea collection from the Greater Sunda Island of Sumatra, approximately 1700 km from the type locality. This is the third specimen known for *Lentistivalius philippinensis* and the female sex remains undescribed. Such a disjunct distribution is indicative of the dearth of ectoparasite collections throughout the Malay Archipelago. A host voucher specimen of “*Rattus rattus diardii*” is not available to verify the recorded field identification. Wilson and Reeder (2005) included “diardii” as a synonym of *Rattus tanezumi* Temminck, a species widely introduced throughout insular Southeast Asia, including the Greater and Lesser Sunda Islands and the Molucca Islands (Flannery 1995). *Rattus tanezumi* might be considered relevant in the apparent dissemination of *Lentistivalius philippinensis* to Sumatra or from Sumatra to the Philippines. In addition, although *Crocidura grayi* is restricted to the island of Luzon, the importance of other species of *Crocidura* across the region may also account for the broader distribution on islands outside of the Philippines, such as Sumatra. Traub (1972a) noted, “… the remarkable facility of this genus [*Lentistivalius*] to adapt to a broad variety of hosts, in widely separated areas”, a statement supported by the various species of *Lentistivalius* that are found on birds, shrews, and murid rodents. Flea collections throughout the Malay Archipelago are drastically lacking. Future collections and studies of fleas and other ectoparasites are badly needed in Southeast Asia and the insular regions of the Malay Archipelago, especially with the alarmingly rapid destruction of habitat and loss of mammalian host species.

##### Medwayella independencia

Hastriter & Bush sp. n.

http://zoobank.org/C1638809-5CB9-4203-8A6A-549C210C4B2A

http://species-id.net/wiki/Medwayella_independencia

###### Type material.

Philippines, Mindanao Island, village Lunutan (08.6959°N, 125.0259°E), Mt. Lumont, 1236 m, *Urogale everetti* (Thomas) ♂ (host # KUMNH-168413, NCA-307), 4 VII 2012, E. DiBlasi, holotype ♂, allotype ♀ (parasite # P-5525) (CMNH), 1♂ paratype (dissection), (BYUC); same data as holotype except *Urogale everetti* ♂ (host # KUMNH-168696, NCA-382), 10 VII 2012, 1♂ paratype (parasite # P-5657) (CMNH), 1♀ paratype (BYUC), *Urogale everetti* ♂ (host # KUMNH-168719, NCA-383), 10 VII 2012, 2♂ paratypes (DNA F-345) (parasite # P-5658) (BYUC), and *Urogale everetti* ♂ (host # KUMNH-168372, NCA-351), 6 VII 2012, 1♂ paratype (parasite # P-5585) (CMNH).

###### Diagnosis.

Males are distinguished from all other species of *Medwayella* by 1) the deep sinus (as deep as width) below the subapical lobe on the dorsal surface of the distal arm of the S-IX, 2) the very long upper arm of the securifer of Ford’s sclerite, 3) the lower arm of the securifer of Ford’s sclerite is lobular, 4) the quasi crochet is distinctly squared at its apex, and 5) the presence of a long, slender recurved spur on the dorsal surface at the basal third of the sclerotized inner tube (Figs 6–7). Females are not separable from those of other species of *Medwayella*.

###### Description.

**Figures 1–4.**
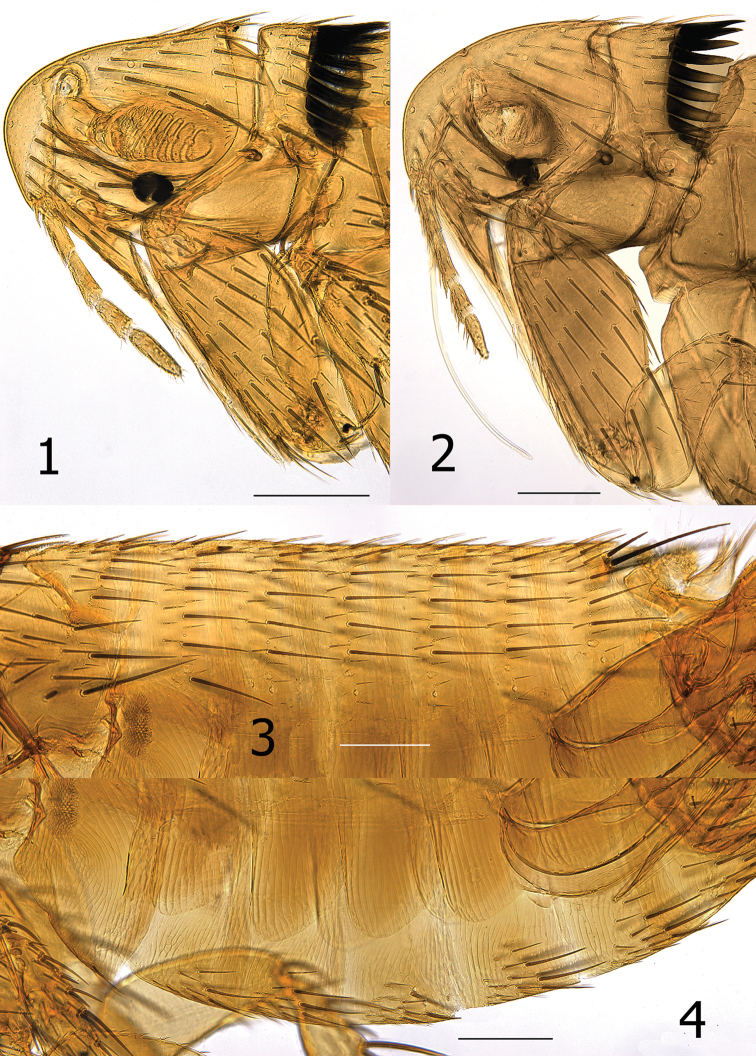
*Medwayella independencia* sp. n. **1** Head and pronotum, male holotype (P-5525) **2** Head and pronotum, female paratype (P-5657) **3** Abdominal tergites, male holotype (P-5525) **4** Abdominal sterna, male holotype (P-5525). (Scale: **1–4** = 200 µm).

**Figures 5–8.**
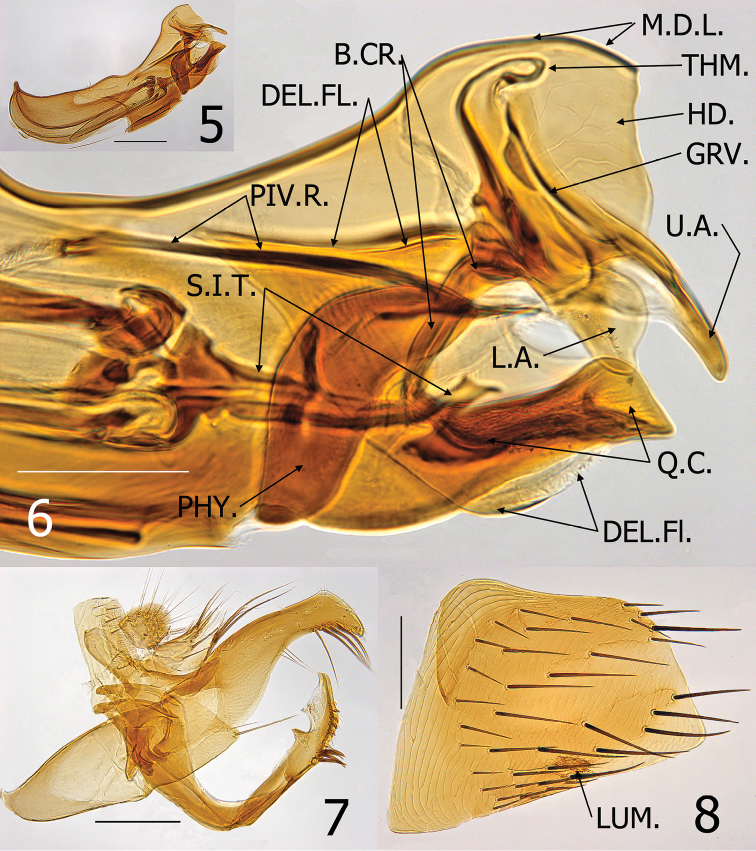
*Medwayella independencia* sp. n. male paratype (P-5525). **5** Aedeagus **6** Enlargement of apex of aedeagus (B.CR. = body of crochet, DEL.FL = deltoid flap of hood of aedeagus, GRV. = groove-like structure of Ford’s sclerite, HD. = hood of aedeagus, L.A. = lower arm of securifer, M.D.L. = median dorsal lobe of aedeagus, PHY. = phylax, PIV.R. = pivotal ridge of phylax, Q.C. = quasi-crochet, S.I.T. = sclerotized inner tube, THM. = thumb-like apex of alpha-portion of Ford’s sclerite, U.A. = upper arm of securifer **7** Basimere, telomere, and S-IX **8** Eighth sternum, LUM. = lumacaudate process. (Scale: **5, 7–8**= 200 µm; **6** = 100 µm).

**Figures 9–12.**
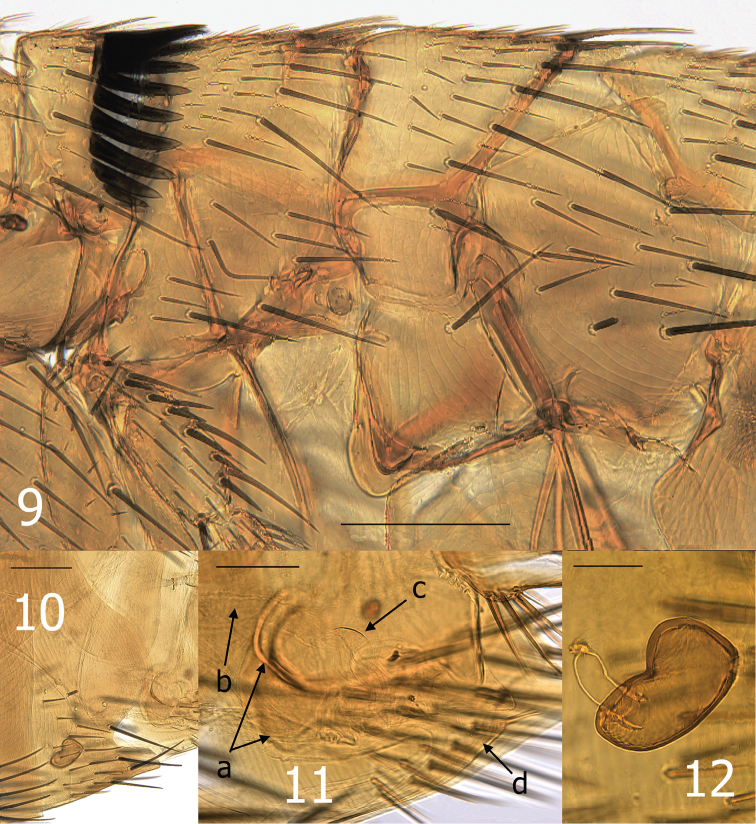
*Medwayella independencia*, sp. n. **9** Thorax, holotype male (P-5525) **10** Seventh sternum, female paratype (P-5657) **11** Vaginal canal, female allotype, a = mesal tanned ridge of T-VIII, b = dilated portion of duct of spermatheca, c = sclerotized ventral wall of perula, d = eighth sternum **12** Spermatheca, female allotype. (Scale: **9–10** = 200 µm, **11** = 100 µm, **12** = 50 µm).

Numbers of setae represent one side of the flea unless otherwise stated. Head (Figs 1–2). Frons evenly rounded from antennal falx to oral angle; falx slightly indicated in male, more so and longer in female. Two placoid discs in pre-antennal area and three in occipital area. Pre-antennal area with four vertical rows of long setae (6, 3, 4, and 3); second row in female with upper setae long and lower three much weaker. A small patch of 4–5 setae between second and third rows of setae. Area anterior to first (front) row of setae delineated by minute punctuations (differing from smooth area behind anterior row of setae). Maxillary palpus 4 segmented; penultimate segment shortest. Maxillae long and acutely sharp at apex. Labial palpus of 5 segments extended to middle of trochanter: apical segment longest. Antennal scape with oblique row of 5–6 short setae, pedicel fringed with 5 short setae, none extending onto clavus. Clavus of male symmetrical, extended to margin of head. Clavus of female short and asymmetrical, only 6 visible segments with basal 4 apparently fused. Numerous setulae along dorsal margin of antennal fossa; group of multiple setulae at each end of marginal row of setulae. Eye large, darkly pigmented, sinuate, fused to gena, and dorsum bulging into antennal fossa. Occipital area with four rows of setae (male: 5, 7, 1 and 7 with intercalaries; female: 6, 7, 1 and 6 with intercalaries). Intercalaries are extended posteriad to that of posterior row in lower setae. Thorax (Fig. 9). Pronotal ctenidia longer than pronotum in male, sub-equal in female; both sexes with total of 18 regular ctenidia plus the last spine about ¼ the length of more dorsal spines. Lateral pronotal ctenidia slightly curved (concave upper, convex lower margins). Pronotum with two rows of setae; main row complete, anterior row incomplete. Lowest seta of main row twice length of other setae. Prosternosome without notch for cervical link plate. First thoracic link plate robust and housing spiracle; second thoracic link plate with ventral sclerite protruding down from anterior apex (fused with wall of mesepimeron). Meso- and metanota each with three rows of setae; main row with intercalaries. Metanotum with single pseudoseta under metanotal collar; unusual among other taxa in that the seta is stout and spine-like. Mesepisternum with three closely spaced short setae and one long seta. Mesepimeron with 7 setae in male, 5 in female; each sex with alveoli of one seta situated directly over spiracle (spiracle opens beneath pleuron of mesepimeron. Pleural rod bifurcate. Lateral metanotal area separated from metepimeron by suture; metepimeron with single long seta. Pleural arch and pleural ridge robust. Squamulum present; long. Metasternum rounded. Setae on metepimeron variable in number and arrangement: 10–16 long setae and 1–7 small setae in male; 11–12 long setae and 4–8 small setae in female. Differs from side to side in same specimen. All setae below level of spiracle on metepimeron. Legs. Fore coxa with numerous scattered setae on upper ⅓; setae on lower ⅔ arranged in two rows more or less with 6–7 setae in each row. Suture of mesocoxa complete. Setae confined to anterior margin and apical ½ of meso- and meta-coxae. Ten to 12 small lateral setae on fore femur. Single long stout seta at femoral-tibial joint of fore tibia; short lateral spiniform seta and long stout mesal seta at femoral-tibial joints of meso- and metatibiae. All tibiae with seven dorsal notches: setae per notch of fore tibia (2, 2, 2, 2, 2, 2, 2), meso- and metatibiae (2, 2, 2, 2, 2, 2, 3); notch three with one of two setae minute. Lateral surface of all tibiae clad with numerous setae. First tarsal segment of fore leg with fringe of 4 large setae on caudal margin. Setae on basal portion of first tarsi of meso- and metatarsi small and un-pigmented; more distal setae distinctly more spiniform and darkly pigmented. Distotarsomeres with six lateral plantar bristles; basal three stouter than distal three pairs. Fore and meso-distotarsomeres with four spiniform pre-apical plantar bristles and meta-distotarsomere with two stout sharp pre-apical plantar bristles in male; female with two pre-apical plantar bristles on each distotarsomere. Mesal surface of tarsal claws on hind leg serrate; claws of anterior legs not serrate. All claws with stout basal lobe. Unmodified Abdominal Segments, male (Figs 3–4). Tergites I–VII each with three rows setae; T-II–V with single spinelet per side. Single seta of main row of each tergite below level of spiracles II–VII. Two antesensilial bristles; mesal bristle half length of lateral. Tergum VIII reduced with 4 small setae and large eighth abdominal spiracle. Sensilium convexly globular; brush-like setae surrounding 16–18 sensilial pits. Base of sensilium with sclerotized projection bearing several minute setae at apex. Dorsal anal lobe pointed with 5–6 long setae. Ventral anal lobe conical with two long setae near apex. Sternum I with 2 small ventral setae; group of multiple minute leuco discs on lateral surface. Sterna III–VII with main row of three long setae; anterior scattered setae. Modified Abdominal Segments, male (Figs 5–8). Dorsal and ventral margins of manubrium nearly parallel; upturned at apex. Stiva of telomere developed; with marginal group of four stout long setae ventrad to stiva and three smaller setae below main group. Telomere with oblique lateral row of minute setae. Dorso-anterior angle of telomere with two un-pigmented small spiniform setae. Base of telomere twice its narrowest width near group of four setae. Basimere with single triangular lobe bearing one long apical acetabular bristle and a smaller bristle dorsal to apex. Base of telomere extended to condyle hinging at base of fulcral sclerite. Proximal arm of S-IX expanded at apex; connected to and integrally associated with fulcral sclerite and base of telomere. Distal arm of S-IX, hyaline along basal dorsal margin with minute spicules. Similar hyaline spiculated area on dorsal surface pre-apically. Spiculated area subtended by a sinus that is as deep as wide. Subapical lobe below sinus pointed with 3–4 caudally directed setae. Ventro-apical margin with five short spiniform setae; medial to these are 6–7 small setae, subtended by two long, darkly pigmented setae. The lower ventral portion of the distal arm without setae; paired distal arms are fused from base to near apex. Sternum VIII quadrate and oblique at apex; ~40 long lateral setae. Lumacaudate process mesal to S-VIII with numerous short, light spiniform setae and one longer darkly pigmented spiniform seta. Median lamella of aedeagus broader than lateral lamella; proximal laminar margin deeply excised from short crescent sclerite to half the distance to the apex of the aedeagal apodeme. Aedeagal apodeme with slight convexity at mid dorsal area; margin sclerotized to arched median dorsal lobe near apex. Hood extended laterally to envelop Ford’s sclerite. Fords sclerite well developed with a caudally directed thumb-like process, with elongate thin upper arm of securifer and lobular lower arm of securifer. Deltoid flap covers sclerotized inner tube, large phylax, and quasi crochet. Sclerotized inner tube long, narrow, straight ribbed at apical ¼ with minute backward pointing spines. A long spur is present on the dorsal surface on the basal ⅓ of the sclerotized inner tube. Pivotal ridge appears fused with dorsal margin of phylax; body of quasi crochet fused with ventral margin of Ford’s sclerite. Quasi crochet blunt at apex; dorso-apical angle rounded, ventro-apical angle moderately acutely angled. Penis rods barely reaching apex of aedeagal apodeme. Ventral wall of aedeagal pouch heavily sclerotized. Unmodified Abdominal Segments, female. Tergites I–VI indistinguishable from male, except T-II with one seta below level of spiracle, T-III–V with two or three, and T-VI with one. Mesal antesensilial bristle half length of lateral bristle (opposite of male). Triangular lobe on margin of T-VII immediately below antesensilial setae; bearing single long seta almost contiguous with lateral antesensilial seta. Lobe extends between pairs of antesensilial bristles; with two stout setae between lobe and mesal antesensilial bristle. Lateral surface S-II with group of four to eight short setae; ventral margin with single setae. Sterna III–V with four setae in main rows, S-VI with five setae in main row and numerous scattered setae anterior to main rows. Modified Abdominal Segments, female (Figs 10–12). Tergum VIII with six to eight setae anterior to “L” shaped eighth spiracle. Tergum VIII large, laterally expanded with caudal triangular process; four marginal long setae dorsal to process and main row of eight long setae sub-marginally with scattered anterior smaller setae. Internal incrassation present on T-VIII. Sternum VII with broad lobe on caudal margin subtended by deep sinus and ventral truncate lobe. Three long setae below sinus on ventral lobe (one out of line) and two similar sized setae above sinus; row of seven or eight medium setae anterior to main row with additional scattered smaller setae anterior to these. Convex sensilium with ventral margin nearly contiguous with dorsal anal lobe; with 16 sensilial pits. Dorsal anal lobe with two very long setae on each side of anal stylet; several smaller setae anterior to base of anal stylet. Anal stylet with single robust seta; two minute setae at base of seta. Ventral anal lobe with basal lobe bearing about six long setae and apical pair of long setae; these interrupted by space without setae. Sternum VIII reduced; narrowing from base to pointed apex that bears several un-pigmented stout setae. Hilla of spermatheca inserted into bulga and perpendicular to linear axis of bulga. Proximal end of bulga wider than distal portion. Perula with expanded hood; ventral portion slightly sclerotized. Duct of spermatheca dilated from perula of bursa for distance about equal to length of bulga. Bursa copulatrix and *glandula vaginalis* expanded, *duplacatura vaginalis* distinctly recurved.

###### Dimensions.

Male holotype: 3.0 mm, male average: 3.3 mm (n = 3); range: 3.0–3.5 mm. Female average: 3.7 mm (n = 2); range: 3.3–4.6 mm).

###### Etymology.

The date on which the holotype was collected was the fourth of July, a national holiday in the United States commemorating the independence of the United States from Great Britain in 1776, thus the specific epithet *independencia*.

###### Remarks.

This new species was collected from the Mindanao Tree-Shrew, *Urogale everetti*. *Urogale everetti* is restricted to the most southerly Philippine islands of Minadanao, Dinagat, and Siargao (Wilson and Reeder 2005). This tupaiid is diurnal with arboreal and terrestrial habits much like North American sciurids. Like most species of *Medwayella*, *Medwayella independencia* occurs on a member of the family Tupaiidae. This is the first published record of *Medwayella* in the Philippines, although Traub (1972a, 1972b) alluded to two new species of *Medwayella* on Mindanao Island and a third on Palawan Island. Those were never published and the repositories of specimens from Mindanao are unknown, however, a pair of *Medwayella robinsoni* from Palawan are reported herein (see below). It is unlikely that any of the specimens to which Traub alluded were *Medwayella independencia* because he stated that he knew of no unique fleas on *Urogale* (Traub, 1972b). The discovery of *Medwayella independencia* on four different males of *Urogale everetti* in the same locality, while absent on other mammalian hosts, would indicate a significant association. Additional collecting is required to substantiate this relationship.

An additional single male and female *Medwayella* was discovered among unidentified material loaned to the author (MWH) by the National Museum of Natural History, Washington, DC. Label data on this single slide included: “Chicago Nat. Hist. Museum, RT 6492, CNHM 2612-2625, ex *Sciurus* [*Sundasciurus hoogstraali* (Sanborn), the Busuanga Squirrel, FMNH Mammal catalog numbers 63077-63095, Pers. Comm. with Lawrence R. Heaney, FMNH], locality: Dimaniang, Busuanga Island, Palawan Province [Philippines, part of the Calamian group], 17 III 47, leg. H. Hoogstraal, Philippines Expedition”. The identification label indicated: “*Stivalius robinsoni*” det. R. Traub, 1949” and inscribed in pencil was “sp. n. – Traub in ms”. To date, there are four published subspecific populations of *Medwayella robinsoni* but none occur as far northeast as the Philippine islands. These two specimens represent a subspecific population of *Medwayella robinsoni*. There are few morphological differences in the current published subspecific populations of *Medwayella robinsoni*. Therefore, adding an additional subspecies is of little taxonomic value, but is reported here to further expand the range of the genus as a supplement to the new *Medwayella* taxon described herein.

**Figure 13.**
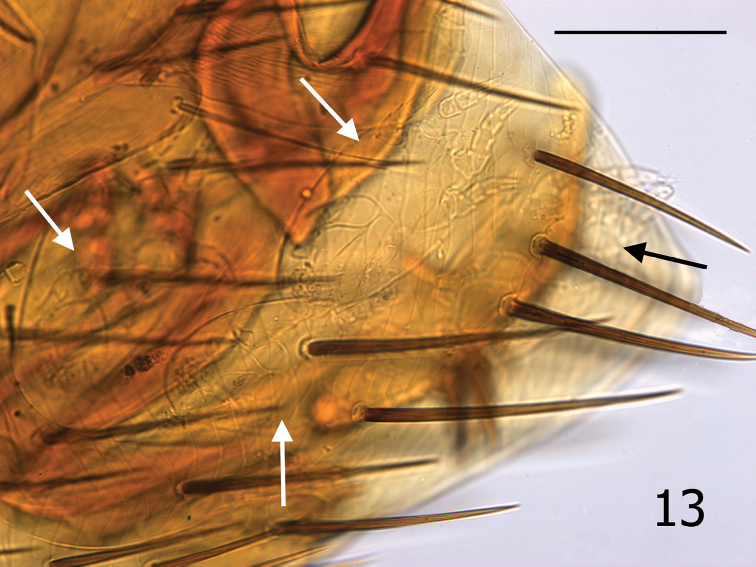
*Medwayella independencia*, sp. n., holotype male (P-5525) terminal segments illustrating four mites beneath T-VIII (see arrows). (Scale: 400 µm).

Phoretic astigmatid mites were noted on three of the five whole mounted specimens of *Medwayella independencia*. Phoresy among mites is a well-known behavior (Houck and OConnor 1991, Bajerlein et al. 2013) and involves numerous accounts of the deutonymphs infesting fleas (Fain and Beaucournu 1993). The bionomics of the twelve genera representing three families (Acaridae, Histiostomatidae and Winterschmidtiidae) that infest fleas (Fain and Beaucournu 1993) are poorly understood. All mites infesting *Medwayella independencia* appear to represent the same species. These mites are tentatively identified as the genus *Psylloglyphus* (family Winterschmidtiidae) (Personal Comm. Dr. Barry M. OConnor, University of Michigan, Ann Arbor, MI, based on his observation of image, Fig. 13). Fain and Beaucournu (1976) described *Psylloglyphus maculatus* from *Medwayella robinsoni* ssp. collected from *Tupaia glis* Diard and Duvaucel from Selangor, Malaysia. Our record is the second documentation of a phoretic mite found on the genus *Medwayella*.

The senior author (MWH) has observed phoretic hypopial mites over 40 years on many different flea species; however, it is unusual for them to situate themselves under the terminal tergite (T-VIII) of male fleas as are the four mites seen in the male holotype (Fig. 13). An additional mite (not shown in Fig. 13) was present under T-VI of the same specimen. The mites usually attach themselves under more anterior sclerites. Mites were also present under T-VIII of a second male, and one female had one mite each under T-VI and S-VII, and two mites under S-V. The usual absence of mites under the T-VIII of males may be a reflection of the lack of protection afforded in this anatomical position. Deutonymphal hypopi possess sucker-like organelles on their venter that aid in affixing them to the underside of the overlapping abdominal sclerites of fleas. The T-VIII of species of *Medwayella* is large and expands broadly, enveloping the aedeagus. During flea copulation, the extreme movement of the aedeagus would potentially produce an unstable environment for the attached mites by mechanically dislodging them. Phoretic mites locate beneath the abdominal sclerites of fleas as an adaptive strategy to avoid being dislodged during host grooming and flea locomotion. Phoretic mites of fleas have not been reported to attach to any other anatomical sites on the fleas other than under the abdominal segments, nor do they ever appear to feed, or attach with their mouthparts. The mobility of these minute phoretic mites is unmistakably very limited; therefore, attaching to a flea is a means to increase their distribution and survival.

OConnor and Pfaffenberger (1987) concluded that some phoretic mite species were highly specific for certain flea species, while others appeared less selective in their choice of flea host species. Subsequently, Schwan (1993) demonstrated a high level of specificity of *Psylloglyphus uilenbergi* Fain occurring on two species of fleas, and of *Paraceroglyphus xenopsylla* Fain and Schwan on a single flea species. The specificity of hypopial mites to a particular flea species may facilitate their attainment of an optimal environment (in a different nest of the flea’s preferred host species and/or assuring the post-deutonymphal stages an optimal nest in which to develop) than otherwise might be possible. Fain and Beaucournu (1993) listed 83 flea species (excluding subspecies) that harbored astigmatid phoretic mites. Noteworthy are the distributions of the three mite families and the associations with their fleas: 1) the single genus (*Psylloglyphus*) in the family Winterschmidtiidae is found only in the southern hemisphere, and their flea host families are also exclusive to these areas; 2) of the two genera in the family Histiostomatidae, one genus is limited to continental Africa, and the second genus is limited to Europe, while one host flea genus (*Ctenophthalmus*) occurs in both Europe and Africa; and 3) the distributions of all genera of the family Acaridae are restricted to the northern hemisphere, and their flea host families are also limited to the northern hemisphere, with the exception of two species. One of these [*Notiopsylla kerguelensis kerguelensis* (Taschenberg)] is found on sea birds with extensive flight ranges, and the other is a cosmopolitan flea species [*Xenopsylla cheopis* (Rothschild)] found commonly on commensal rodents. *Psylloglyphus* mite species in our report appear to be specific to *Medwayella* (a southern hemisphere flea), a trend that coincides with the observations reported by Fain and Beaucournu (1993) for numerous cases of mite phoresy on fleas. It is not known whether fleas have physiological or behavioral defenses against these phoretic mites; however, this is a rich system of potentially co-evolving relationships between mites and fleas that warrants further study. OConnor (1982) provided a more thorough discussion of the evolutionary ecology of astigmatid mites. Although OConnor and Pfaffenberger (1987) and Fain and Beaucournu (1993) provided numerous bibliographic references regarding the studies of phoretic and hypopial astigmatid mites, there remains a large gap in our knowledge of the bionomics and degrees of specificity of phoretic mites that occur on many more species of fleas than are indicated in the literature. Fleas harboring phoretic mites observed in future studies should be placed in ethanol, and provided to a mite specialist for study. Such studies would enhance our understanding of the potential co-evolutionary trends of phoretic mites and their host fleas relative to the biology of each association that occurs in the nests of various mammalian or avian host species.

The following key is inclusive of all known male *Medwayella* taxa. For female specimens, readers are referred to the key (with supporting illustrations) by Traub (1972a: 265–269). Taxa described subsequent to Traub (1972a) included *Medwayella independencia*, *Medwayella rubrisciurae* Durden and Beaucournu, *Medwayella pheifferi* Beaucournu and Wells, *Medwayella sabahae* Beaucournu and Wells, and *Medwayella traubiana* Beaucournu and Wells. In addition, *Medwayella cambodia* (Klein) was transferred to *Medwayella* by Mardon (1981). Of these six additional species, the female sex is described only for *Medwayella independencia*. The female sex of *Medwayella independencia* keys out to couplet 20 (Traub 1972a) (*Medwayella dryadosa* Traub and *Medwayella veruta* Traub) but cannot be distinguished from either of these species.

##### Key to males of *Medwayella*

**Table:** 

1	Labial palpus extends to apex of trochanter	*Medwayella calcarata*
1’	Labial palpus extends at most to apex of fore coxa	2
2(1’)	Stiva short, hidden by distal fringe of stout setae on ventro-apical margin	*Medwayella rubrisciurae*
2’	Stiva of various lengths but not hidden by distal fringe of setae	3
3(2’)	Ventro-apical margin of distal arm of ninth sternum without marked sclerotization or sub-marginal delineation	4
3’	Ventro-apical margin with distinct sclerotized margin, or sub-marginal delineation	13
4(3)	Sub-apical dorsal notch or sinus present on distal arm of ninth sternum	5
4’	Sub-apical dorsal notch or sinus absent	8
5(4)	Dorsal portion of Ford’s sclerite with distinct thumb-like process	6
5’	Ford’s sclerite lacking a thumb-like process	7
6(5)	Sub-apical lobe of distal arm of ninth sternum sharply pointed; sub-apical sinus shallow	*Medwayella sabahae*
6’	Sub-apical lobe triangular but not sharply tapered to a point; with deep sub-apical sinus between sup-apical lobe and apical lobe	*Medwayella robinsoni* ssp.
7(5’)	Apex of distal arm of ninth sternum tapering to rounded point; subtending sinus distinctly wider than deep	*Medwayella traubiana*
7’	Apex of distal arm bluntly rounded or truncate; subtending sinus hardly wider than deep	*Medwayella angustata*
8(4’)	Distal fringe of long setae on ventro-apical margin of telomere shifted towards apex and arising from near apex of stiva; this group of setae subtended by deep sinus between distal fringe of setae and more proximal group of ventral setae (depth of sinus greater than width of telomere at deepest point of sinus)	*Medwayella rhaeba*
8’	Distal fringe of long setae some distance from apex of stiva; if placement is close to stiva, then sinus is more shallow than width of telomere	9
9(8’)	Thumb-like process present on dorsal portion of Ford’s sclerite	10
9’	Ford’s sclerite lacking thumb-like process	12
10(9)	Sub-apical lobe of distal arm of ninth sternum sharply pointed and directed downward; apical lobe broadly rounded	*Medwayella arcuata*
10’	Sub-apical lobe rounded, not sharp; apical lobe round at apex but sides more parallel and more slender	11
11(10’)	Apical lobe of distal arm of ninth sternum extended well over sub-apical lobe (beyond, appearing to arch over); telomere narrower at base than other more distal portions of process	*Medwayella javana*
11’	Apical lobe shorter; not arching over and beyond sub-apical lobe; telomere much broader basally than more distal portions	*Medwayella cambodia*
12(9’)	Sub-apical lobe of distal arm of ninth sternum reduced to flattened bulge; distance from apex of sub-apical lobe to closest point on ventral margin less than distance to apex of apical lobe	*Medwayella pfeifferi*
12’	Sub-apical lobe triangular (as tall as wide); distance from apex of sup-apical lobe to ventral margin about equal distance to apical lobe	*Medwayella phangi* ssp.
13(3’)	Margin between sub-apical lobe and apical lobe of ninth sternum with sinus as deep as wide	*Medwayella dryadosa*
13’	Margin without sinus, or sinus much broader than deep	14
14(13’)	Groove formed by sclerotized margins of upper and lower arms of securifers of Ford’s sclerite present	*Medwayella independencia* sp. n.
14’	Groove of Ford’s sclerite absent or unapparent	15
15(14’)	Sub-apical lobe of ninth sternum hardly visible (indicated by shallow bulge); thumb-like process present on dorsal portion of Ford’s sclerite	*Medwayella thurmani*
15’	Sub-apical lobe well developed; thumb-like process absent	16
16(15)	Sub-apical lobe of ninth sternum narrow and sharply pointed	17
16’	Sub-apical lobe well developed, but broadly triangular and rounded at apex	*Medwayella limi*
17(16)	Telomere short and broad, hardly more than 3 times as long (above apex of basimere) as broad at narrowest level (immediately below distal fringe of setae on ventral margin)	*Medwayella loncha*
17’	Telomere at least 3.75 times as long as broad (at narrowest level)	18
18(17’)	Lower arm of securifer as broad as long; distal arm of ninth sternum much broader below sub-apical lobe than above it	*Medwayella veruta*
18’	Lower arm of securifer not broad but vermiform; width of distal arm about same above as below sub-apical lobe	*Medwayella batibacula*

## Supplementary Material

XML Treatment for Macrostylophora sp.

XML Treatment for Thaumapsylla breviceps orientalis

XML Treatment for Thaumapsylla longiforceps

XML Treatment for Lentistivalius philippinensis

XML Treatment for Medwayella independencia
